# Multi-Class Classification Methods for EEG Signals of Lower-Limb Rehabilitation Movements

**DOI:** 10.3390/biomimetics10070452

**Published:** 2025-07-09

**Authors:** Shuangling Ma, Zijie Situ, Xiaobo Peng, Zhangyang Li, Ying Huang

**Affiliations:** 1 College of Mechatronics and Control Engineering, Shenzhen University, Shenzhen 518060, China; 2410095088@email.szu.edu.cn (S.M.); 2021110092@email.szu.edu.cn (Z.S.); 2110292136@email.szu.edu.cn (Z.L.); 2 Shenzhen Key Laboratory of Marine Bioresources and Ecology, College of Life Sciences and Oceanography, Shenzhen University, Shenzhen 518060, China

**Keywords:** MI-EEG signals, four-class classification, common spatial pattern, convolutional neural network, 3D EEG-CNN

## Abstract

Brain–Computer Interfaces (BCIs) enable direct communication between the brain and external devices by decoding motor intentions from EEG signals. However, the existing multi-class classification methods for motor imagery EEG (MI-EEG) signals are hindered by low signal quality and limited accuracy, restricting their practical application. This study focuses on rehabilitation training scenarios, aiming to capture the motor intentions of patients with partial or complete motor impairments (such as stroke survivors) and provide feedforward control commands for exoskeletons. This study developed an EEG acquisition protocol specifically for use with lower-limb rehabilitation motor imagery (MI). It systematically explored preprocessing techniques, feature extraction strategies, and multi-classification algorithms for multi-task MI-EEG signals. A novel 3D EEG convolutional neural network (3D EEG-CNN) that integrates time/frequency features is proposed. Evaluations on a self-collected dataset demonstrated that the proposed model achieved a peak classification accuracy of 66.32%, substantially outperforming conventional approaches and demonstrating notable progress in the multi-class classification of lower-limb motor imagery tasks.

## 1. Introduction

BCIs facilitate the direct control of external devices by decoding brain activity, integrating software and hardware systems, and have drawn considerable attention globally [1]. Extensive research efforts have focused on developing experimental paradigms and acquiring EEG signals under various motor and cognitive conditions. These advancements have significantly enhanced EEG-based BCI technologies, especially in clinical applications, providing vital communication channels for individuals with stroke, paralysis, or neuromuscular disorders affecting their lower-limb mobility [2].

Electroencephalography (EEG) is a non-invasive method for recording cortical electrical activity from the scalp, utilizing either dry or wet electrodes to detect neural potentials originating intracranially [3]. Given its safety, affordability, and excellent temporal resolution, EEG has become a predominant technique in BCI research [4]. At present, most methods for recognizing lower-limb MI signals are based on EEG signal classification and identification [5,6]. Most current approaches predominantly employ binary EEG-based classifications, limiting their applicability to real-world motor tasks characterized by greater complexity. The four-class strategy proposed in this study supports a wider range of rehabilitation movement types, making it more aligned with practical application needs. Indeed, multi-class EEG decoding remains a significant challenge and an active area of ongoing research [7]. Notably, compared to upper-limb motor imagery, fewer studies have specifically investigated EEG acquisition protocols and classification models tailored to lower-limb rehabilitation. To contribute toward addressing this gap, the present study systematically explored the entire pipeline, including the experimental setup, signal preprocessing, feature extraction, and model development. The contributions of this work are summarized as follows:

1. An EEG acquisition platform tailored for use in lower-limb rehabilitation MI tasks was established. A hybrid cueing paradigm combining visual symbols with instructional videos was adopted to minimize visual distractions and enhance event-related potentials. EEG data were recorded using a 64-channel cap to achieve comprehensive spatial coverage and high signal fidelity.
2. An automated artifact rejection tool, based on the MNE toolbox and PyQt5 framework, was implemented. By extracting 15 independent components through ICA, this method accurately identified and efficiently removed various artifacts, such as electromyographic (EMG), electrooculographic (EOG), electrocardiographic (ECG), power-line interference, and impedance noise, significantly outperforming traditional manual methods.
3. Comprehensive analyses were conducted to extract motor imagery features across temporal, frequency, and spatial domains. Several enhanced variants of the common spatial pattern (CSP) algorithm were developed, optimizing feature selection for effective multi-class classification using support vector machines (SVMs).
4. A novel convolutional neural network (CNN) model was designed to analyze 3D EEG representations, incorporating both temporal sequences and frequency-domain power spectral densities. This CNN architecture integrates deep convolutional layers, effectively capturing spatial features from EEG channels while minimizing the computational complexity. Additionally, a dual-branch input strategy independently encodes spatial dimensions from EEG topographic maps, subsequently merging them into a unified structure for improved classification performance.

## 2. Related Work

In recent years, there has been a growing interest in the four-class classification of MI EEG signals. Research efforts have primarily focused on two key aspects: the extraction of discriminative features and the development of effective classification algorithms. Common feature extraction techniques include CSP [8], independent component analysis (ICA) [9], and methods based on Riemannian geometry [10,11]. These features are typically classified using machine learning approaches such as support vector machines (SVMs) [12], convolutional neural networks (CNNs) [13], and linear discriminant analysis [14,15]. The current research is primarily dedicated to enhancing these foundational methods to improve the classification accuracy in MI-based BCI systems.

The binary classification of MI EEG signals has become a relatively mature area within BCI research, with numerous studies reporting average accuracies exceeding 90% on publicly available datasets. For instance, Dai et al. [16] proposed a hybrid-scale convolutional neural network for the classification of EEG-based MI signals. By incorporating convolutional channels with multiple kernel sizes, the model enables the parallel extraction of multi-scale features. It achieved average classification accuracies of 91.57% and 87.6% on the BCI Competition IV 2a and 2b datasets, respectively. Zhang et al. [17] proposed an ensemble framework for lower-limb MI-EEG classification, combining wavelet decomposition and a filter bank common spatial pattern (FBCSP) method with deep learning modules including CNNs, multi-head self-attention mechanisms, and temporal convolutional networks (TCNs). Validated through offline and online experiments with a customized lower-limb exoskeleton, the method achieved superior accuracy (up to 60.27% for three-class tasks) compared to models such as EEGNet and ATCNet, demonstrating the effectiveness of integrating interpretable and deep-learned features when developing gait rehabilitation BCIs. Muhammad et al. [18] developed a one-dimensional CNN architecture with standardized convolutional layers, further enhanced through the integration of EEGNet-based 2D CNNs, long short-term memory (LSTM) units, gated recurrent units, and attention mechanisms. Their model achieved an accuracy of 96.4% in a binary classification task for detecting developmental delay in children, demonstrating robust clinical applicability. Yang et al. [19] proposed a graph-based long short-term memory convolutional neural network, which integrates graph convolutional networks, spatial convolution, and LSTM modules to jointly extract temporal, spectral, and spatial features. Their method achieved a binary classification accuracy of 78.65% on the MEG-BCI dataset. Chunxiao Han et al. [20] proposed a 1D-CNN-LSTM model for classifying lower-limb MI-EEG signals. EEG data were recorded from 12 subjects performing the imagined flexion and extension of both legs. The model extracted local temporal features using a 1D-CNN and captured long-term dependencies using LSTM. It achieved an average offline classification accuracy of 63.75%, outperforming traditional methods such as CSP-SVM and FBCSP-SVM.

Beyond binary classification, recent years have witnessed a growing body of research focusing on the multi-class classification of MI-EEG signals. Priyanshu et al. [21] proposed a two-stage classification framework based on a Transformer architecture. In the first stage, spatial–temporal features were extracted using a combination of parallel convolutional EEGNet modules, multi-head attention mechanisms, and depthwise temporal convolutional networks. In the second stage, additional and embedded features extracted during the first stage were used to train a TabNet classifier. This method achieved an average classification accuracy of 88.5% on the BCI Competition IV dataset 2a (BCI-IV-2a dataset). Yang et al. [22] introduced a multi-scale fusion convolutional neural network for four-class motor imagery classification. Building upon the original EEGNet architecture, they incorporated attention modules and 2D spatial convolution layers to construct networks at multiple scales and fuse them. A multi-head attention mechanism was used to emphasize the salient features, while 2D spatial convolutions improved feature discrimination and expanded the receptive field. The proposed model achieved average classification accuracies of 87.16% on the BCI-IV-2a dataset and 94.43% on the High-Gamma dataset. Wang et al. [23] proposed a multi-level spatial feature extraction module for BCI applications. The module consists of two stages: in the local feature extraction stage, EEG electrodes are divided into spatial regions, and dedicated convolutional kernels are applied to each region; in the global feature extraction stage, a global convolutional kernel is used to capture spatial-domain features across the entire electrode map. This method achieved a four-class classification accuracy of 89% on the BCI-IV-2a dataset. Liu et al. [24] introduced an automatic feature extraction method for MI-EEG signals based on deep learning. The approach first applies a discrete wavelet transform and denoising to the raw EEG signals, followed by the use of a CNN to extract time-domain features. Simultaneously, spatial-domain features are extracted and enhanced using a multi-head attention mechanism to emphasize critical patterns. Finally, a TCN is employed to extract spatiotemporal features. The method achieved a four-class classification accuracy of 85.53% on the BCI-IV-2a dataset.

Despite the recent progress, the multi-class classification of EEG signals remains a significant challenge. Although prior studies incorporated advanced strategies such as Transformer architectures, attention mechanisms, and multi-scale convolutional networks, the overall classification accuracies typically ranged between 60% and 80%. This suggests that the current approaches remain limited in their ability to capture inter-class distinctions and enhance discriminative precision. The challenge is particularly pronounced in the four-class classification of lower-limb motor imagery, where EEG signals originate from deeper brain regions, exhibit lower amplitudes, and present greater inter-class ambiguity. As a result, the classification performance in lower-limb tasks consistently lags behind that for upper-limb motor imagery. Therefore, effectively extracting discriminative features from lower-limb MI EEG signals and enhancing both the accuracy and robustness of multi-class classification remain pressing and unresolved problems in this field.

## 3. Data Acquisition

### 3.1. Motor Imagery Data Collection During Rehabilitation Tasks

A total of five college students (labeled LLS01 to LLS05) were recruited as participants for this experiment. All were aged between 23 and 25, with normal vision and good health. Each participant was required to practice imagining lower-limb rehabilitation movements for one week prior to the experiment to enhance their motor imagery ability and familiarize themselves with the procedure. The experiments were conducted during daytime hours. The participants were instructed to go to bed before 11:00 PM the night before to ensure optimal mental alertness during the session. The participants were asked to wash their hair and dry it with a hairdryer within one hour before the experiment to ensure a clean scalp. Prior to the experiment, the researcher explained the procedure in detail. The participants were required to complete and sign an informed consent form to ensure they fully understood the experiment.

**Figure 1:**
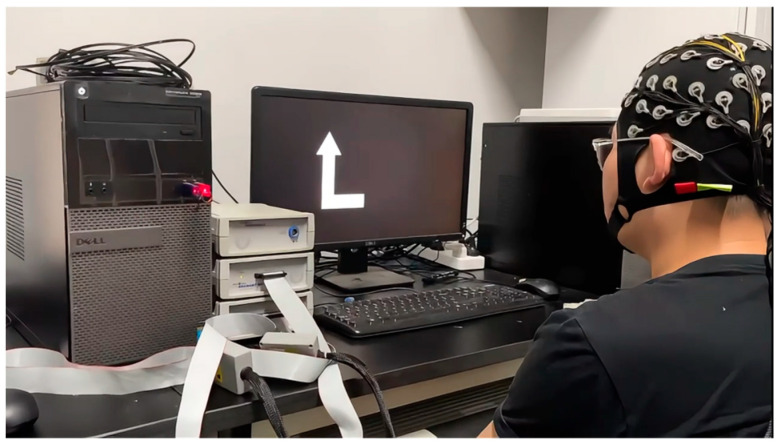
Computer 1 administered the visual imagery cues to the participant.

This study employed two computers to construct a motor imagery experimental platform and adopted a non-invasive method to collect EEG signals from participants during motor imagery tasks. EEG signals were recorded using the BrainProducts EEG acquisition system from Germany, which included a 64-channel electrode cap, a BrainAmp amplifier, and a signal processor. The hardware bandpass filter was configured to the default range of 0.1–250 Hz, and the EEG signals were sampled at 250 Hz, yielding 750 data points for each 3 s motor imagery trial. The software tools used in the system included E-Prime, BrainVision Recorder, and BrainVision Analyzer, which were utilized for designing the experimental paradigm and for EEG signal acquisition and analysis. Using E-Prime, the experimental paradigm was designed to instruct participants via Computer 1 to perform the corresponding motor imagery tasks, as illustrated in Figure 1.

**Figure 2:**
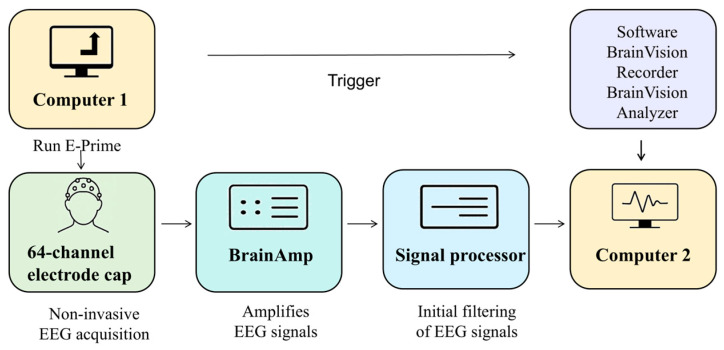
The motor imagery experiment system for lower limbs.

Figure 2 illustrates the procedure for experimental EEG data acquisition. The BrainAmp amplifier amplified the EEG signals collected from the electrode cap at a fixed gain. The signal processor performed the preliminary filtering of the EEG signals. The raw data from all the EEG channels were recorded in real time using BrainVision Recorder and stored on Computer 2. The signals are also displayed on the screen to allow the experimenter to monitor them for abnormalities. A 64-channel electrode cap conforming to the international 10–20 system was used to measure the weak voltage amplitudes at 64 scalp locations.

**Figure 3:**
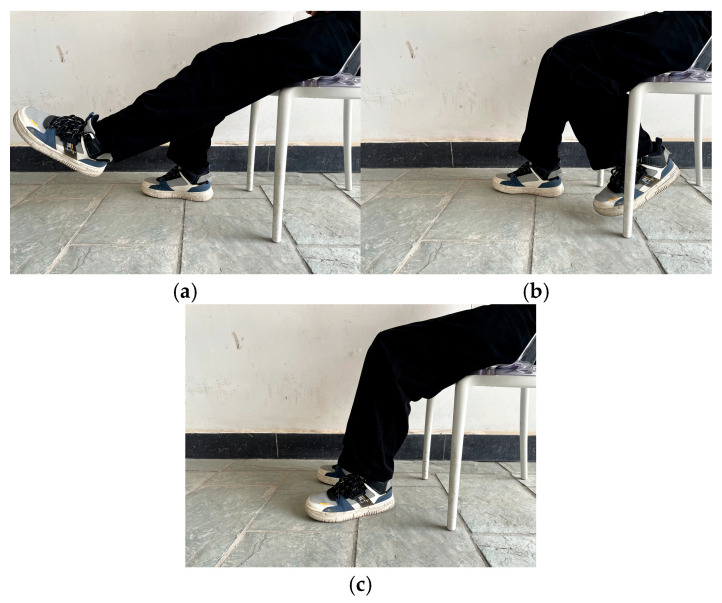
Specific rehabilitation movement states. (**a**) Knee extension state; (**b**) knee flexion state; (**c**) relaxed state.

In this study, four lower-limb rehabilitation movement states were defined: left leg flexion, left leg extension, right leg flexion, and right leg extension. Videos of actual human lower-limb movements were first recorded to serve as experimental cues. Then, visual symbols were used to prompt participants to immediately imagine performing the corresponding movement once, as illustrated in Figure 3. EEG signals were continuously recorded during this period for the subsequent classification of brain activity corresponding to different lower-limb movement states.

**Figure 4:**
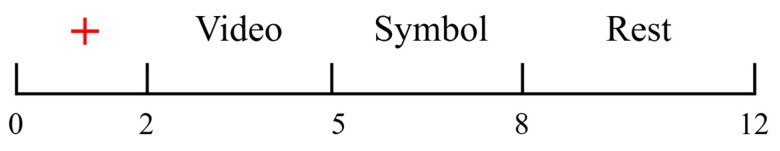
One trial cycle of the experimental protocol.

Each participant completed eight experimental sessions, with each session consisting of 40 individual trials, as illustrated in Figure 4. Each of the four motor imagery tasks was repeated 10 times per session, with each trial lasting 12 s. Before each trial, an instruction screen was displayed. Once ready, the participant could initiate the trial by pressing the space bar. The procedure for a single trial was as follows.

From 0 to 2 s, a red cross appeared at the center of the screen, indicating the upcoming start of the trial and serving to focus the participant’s attention.

From 2 to 5 s, the participant’s lower limbs remained in a relaxed state, as shown in Figure 3c, while a pre-recorded video was played on the screen to indicate the upcoming motor imagery task.

**Figure 5:**
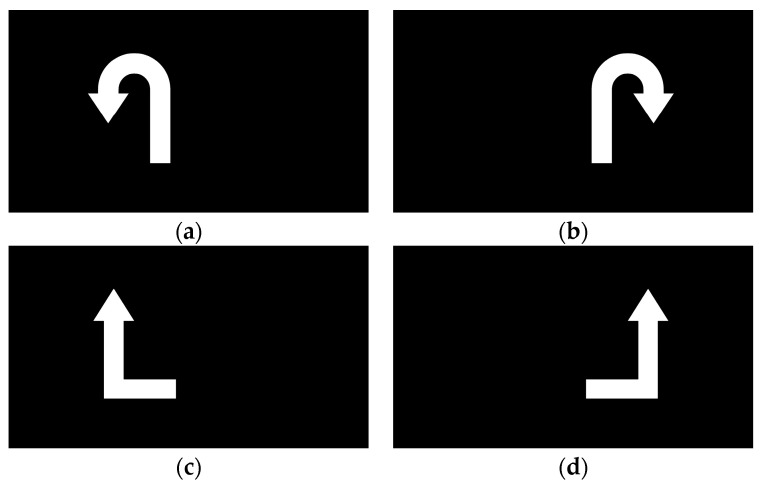
Symbols for four classes of motor imagery states: (**a**) left leg flexion; (**b**) right leg flexion; (**c**) left leg extension; (**d**) right leg extension.

From 5 to 8 s, a visual cue appeared on the screen that corresponded to the movement shown in the preceding video. Upon seeing the cue, the participant immediately began imagining the corresponding lower-limb movement. The visual cues for the four motor imagery tasks (left leg flexion, right leg flexion, left leg extension, right leg extension) are illustrated in Figure 5. At the 5 s mark, the EEG signal was tagged using E-Prime, with each of the four motor imagery tasks assigned a distinct label (S1, S2, S3, and S4). This labeling facilitated the alignment of each EEG segment with the corresponding imagined movement during postprocessing.

From 8 to 12 s, the participant entered a rest period in which they remained in a relaxed state. The participants were instructed to remain relaxed without making large movements, in order to minimize EMG artifacts caused by fatigue.

In addition to the EEG data collected using the experimental platform described above, this study also incorporated the BCI-IV-2a dataset [25], a widely used public benchmark, to facilitate method validation and performance comparisons. The BCI-IV-2a dataset originated from the fourth BCI competition and is a multi-class MI-EEG dataset. It consists of EEG recordings from nine subjects, labeled A01 to A09. The data were recorded using a 22-channel EEG system, with a sampling rate of 250 Hz and a bandpass filter set to 0.5–100 Hz. Each subject performed four types of motor imagery tasks (left hand, right hand, both feet, and tongue) across two sessions conducted on different days. Each session included six runs, with a total of 288 trials per subject (72 trials per class).

### 3.2. EEG Signal Preprocessing

**Figure 6:**
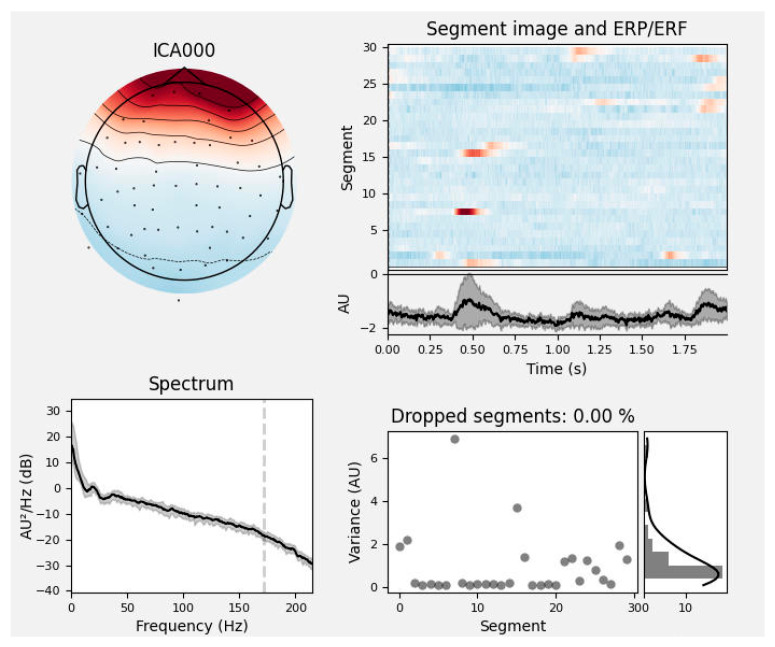
Analysis of ocular artifacts. The top left plot shows the spatial distribution of the ICA component on the scalp; the top right plot presents the time series and ERP/ERF of the component, highlighting the temporal characteristics and the presence of artifacts. The spectrum plot in the lower left shows the power spectral density changing with frequency, indicated by a black line. The lower right plot shows variance in grey dots and the percentage of dropped segments due to artifacts in a black line, with zero percent in this instance.

The raw EEG signals collected during the experiment were often contaminated by various artifacts and noise. In this study, EEG preprocessing was performed using both the EEGLAB toolbox in MATLAB (R2024a) and the MNE-Python package (version 1.6.0), a widely used open-source platform for MEG and EEG data analysis that provides comprehensive tools for filtering, artifact removal, and feature extraction. The preprocessing pipeline consisted of eight main steps: electrode localization, channel selection, re-referencing, filtering, segmentation, bad segment rejection, blind source separation, and artifact removal. During the filtering stage, considering that the EEG signals in the rehabilitation motor imagery dataset were primarily distributed within the theta (4–7 Hz), alpha (8–13 Hz), and beta (14–32 Hz) frequency bands and to suppress low-frequency drifts, a bandpass filter of 0.5–40 Hz was applied. In addition, to eliminate power-line interference caused by the 50 Hz mains electricity in China, a notch filter was employed at 49.9–50.1 Hz. To improve the efficiency of EEG preprocessing, an automatic artifact removal tool was developed in this study. The method is based on the MNE EEG analysis package in Python and employs ICA to identify and remove artifacts from the raw EEG signals. In MNE, the EEG components are categorized into seven types: neural activity, EMG signals, EOG signals, ECG signals, power-line interference, impedance noise, and other signals. The label_components function integrates the multi-domain features of each ICA component—such as its temporal, spectral, and spatial characteristics—to predict the likelihood of each component belonging to a specific artifact class. For example, in Figure 6, the predicted probability that component ICA0 corresponds to an eye movement artifact is 0.99.

**Figure 7:**
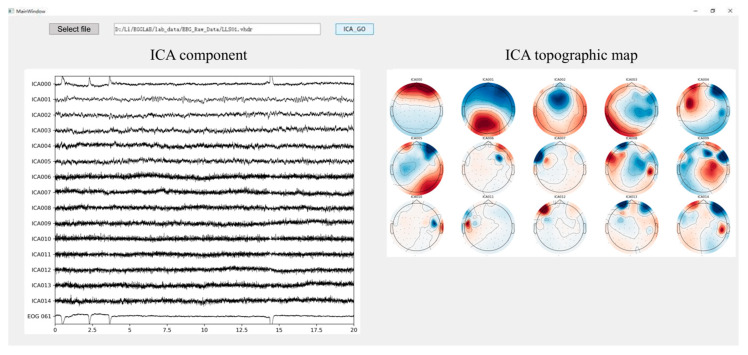
Automatic ICA. ICA topographic maps display scalp topographies using color coding to represent the distribution and intensity of ICA components across the scalp. Warmer colors such as red typically indicate higher activity or amplitude, while cooler colors like blue represent lower activity. The interface includes a button labeled with a term that, in English, means ‘Select File’.

To enhance the automation and efficiency of artifact removal, an interactive tool for automatic artifact removal was developed using PyQt5, leveraging the ICA probability analysis functionality provided by MNE. The user interface is shown in Figure 7. Users simply select the EEG file to be processed via the input field at the top of the interface. In the example shown, EEG data from subject LLS01 in the rehabilitation motor imagery dataset is selected. The tool integrates an embedded ICA component classification algorithm based on MNE, with the number of components preset to 15. It outputs both the time-domain waveforms and scalp topographies of each component. Components predicted to be non-neural signals with a probability greater than 90% are automatically removed. The cleaned EEG signals are saved in .vhdr format and exported automatically.

**Figure 8:**
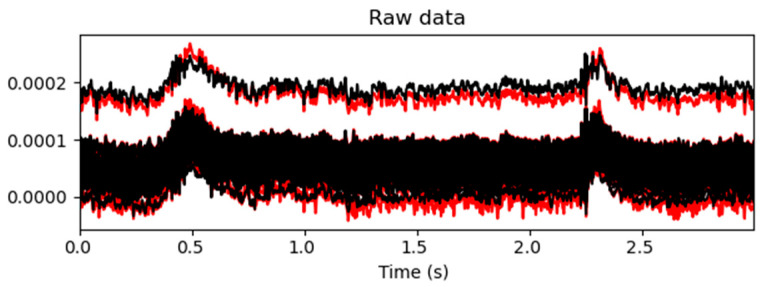
Comparison of the EEG signal quality before and after artifact removal. The upper trace, depicted in black, shows the EEG signal after ICA-based artifact removal. The lower trace, depicted in red, represents the raw EEG signal before processing.

**Figure 9:**
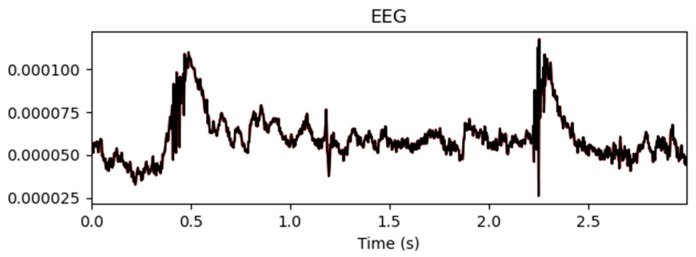
Mean values before and after artifact removal.

A comparison of the EEG time-domain signals before and after artifact removal is shown in Figure 8 and Figure 9. In Figure 8, the red curves represent the EEG signals before artifact removal, while the black curves represent the signals after processing. As can be observed in the figure, at several time points—such as at around 0.5 s and 2.25 s—the red curves exhibited pronounced high-amplitude fluctuations, indicating significant artifact contamination in the raw signals. After automated ICA processing, the amplitude of the fluctuations in the black curves was significantly reduced, resulting in a more stable signal with the evident suppression of physiological artifacts such as eye movements, muscle activity, and cardiac interference. As the figure displays overlaid waveforms from all the channels, the convergence of the black curves suggests that artifact removal not only improved the signal quality at the individual-channel level but also enhanced the overall consistency of the EEG data. This provided a reliable signal foundation for subsequent feature extraction and classification tasks.

Figure 9 shows the mean EEG signal amplitude across all the channels before and after artifact removal. The results indicate that automated ICA effectively suppressed noise and artifact interference while retaining the overall waveform structure of the EEG signals. This ensured a cleaner signal representation, which was beneficial for performing downstream classification tasks.

Comparing our method with the ICA process using the Adjust plugin in the EEGLAB toolbox for MATLAB, the key difference lies in EEGLAB’s reliance on expert-based manual identification. It requires the manual removal of artifact components after the visual inspection of the ICA topographic maps. In contrast, the automated interactive interface developed in this study maintains the classification accuracy while also offering speed and automation. This significantly improves the efficiency of artifact removal and lays the foundation for future real-time EEG processing.

## 4. Methods

This chapter addresses the multi-class classification of EEG signals related to lower-limb motor imagery by proposing a staged research framework consisting of feature extraction, multi-domain representation construction, and deep learning-based classification modeling.

First, based on the spatial and spectral characteristics of EEG signals, variants of the CSP algorithm were designed and improved to enhance the discriminability and robustness of the extracted features. Next, the extracted features were reconstructed into three-dimensional structures to form 3D EEG representations in both the time and frequency domains, preserving the spatial topology across channels. Finally, a multi-channel deep learning model based on CNNs was developed to classify four types of lower-limb motor imagery tasks. This chapter presents the specific methods used in each of these three stages.

### 4.1. EEG Feature Extraction Methods

In non-end-to-end MI-EEG classification algorithms, feature extraction plays a crucial role. It effectively reduces the amount of data that the BCI system needs to process and improves the accuracy of classification.

To comprehensively extract EEG features, researchers typically consider multiple domains, including the time, frequency, time/frequency, and spatial domains. In this study, domain-specific feature extraction methods were applied to left- and right-hand trials in the BCI-IV-2a dataset. The resulting feature vectors were fed into a unified classifier, and five-fold cross-validation was used to evaluate the classification accuracy.

**Figure 10:**
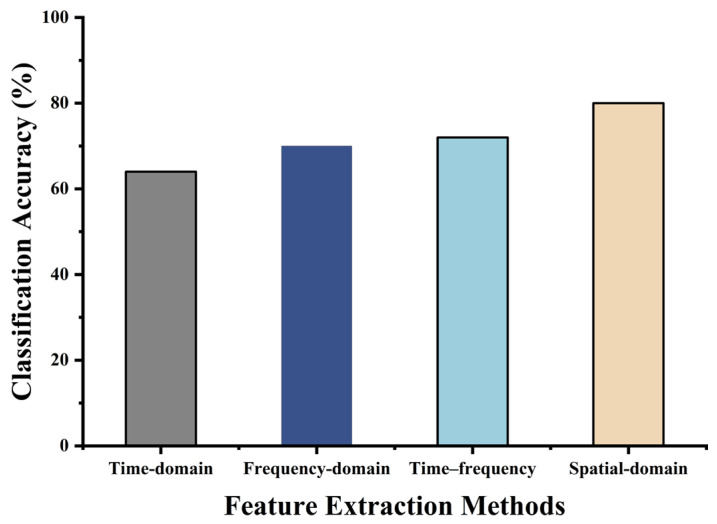
Preliminary comparison of different feature extraction methods.

As shown in Figure 10, the CSP algorithm, which extracts spatial-domain features, exhibited the best performance. Therefore, this section introduces several improvements to the basic CSP algorithm to extract more informative features and provide a better foundation for subsequent multi-class classification.

#### 4.1.1. Weighted-Criterion Common Spatial Pattern (WC-CSP)

The common spatial pattern algorithm, originally introduced by Fukunaga in 1990, is a widely used method for extracting spatial-domain features from EEG signals. It was later applied to EEG signal processing by Ramoser et al. [26].

The design objective of the CSP algorithm is to enhance the discriminability between classes by projecting the original EEG signals into a new spatial domain, rather than preserving all the information from the original signals. As a result, the information distribution within the projection matrix is inherently uneven—different features mapped by the spatial filter, P, contribute unequally to the representation of class-relevant information.

Therefore, in this study, a genetic algorithm was employed to identify the information distribution across the CSP feature matrix, enabling the retention of the most informative features while reducing the overall dimensionality. Due to its global search capabilities and ability to avoid local optima, the genetic algorithm is particularly well suited for feature selection tasks. In the context of EEG spatial filtering, it facilitates the efficient exploration of the search space of spatial filter combinations to select the most discriminative components.

For an EEG training set with C channels, the computed spatial filter matrix P has the dimensions C × C. Based on this, a 1 × C binary-weight gene sequence, W = [w_1_, w_2_, w_3_, …, w_C_], is defined, where each w*_i_* ∈ {0, 1} indicates whether the corresponding spatial filter is retained. The sequence W is converted into a diagonal matrix, diag(W), which is then multiplied with the spatial filter matrix, resulting in a weight-adjusted spatial filter matrix, WP.

(1) $$WP=P⊗\left[\begin{array}{ccc} w_{1} & \ldots & 0\\ \vdots & \ddots & \vdots \\ 0 & \ldots & w_{c} \end{array}\right]$$

**Figure 11:**
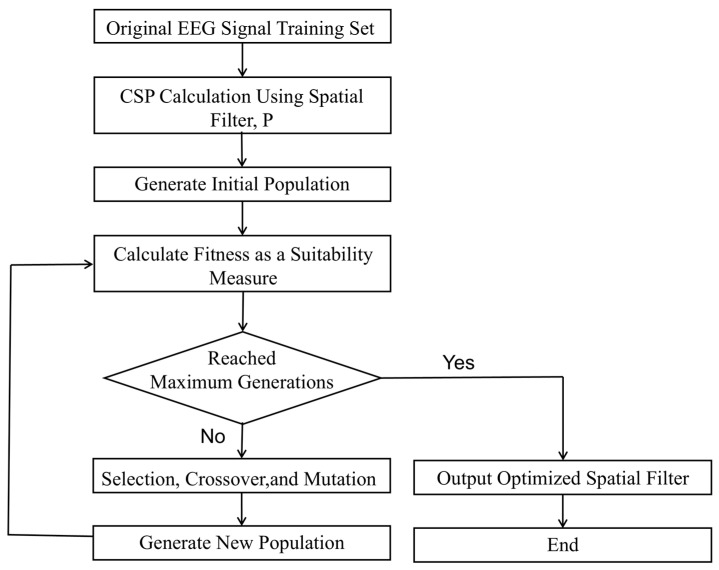
Genetic algorithm-based weight optimization workflow.

The workflow for optimizing the weight matrix using an SVM classifier is illustrated in Figure 11. 

The pseudocode of the weight optimization Algorithm 1 is shown in below.
**Algorithm 1.** Genetic algorithm for feature weight selection.Input: BCI_IV_2a Output: Optimized weight vector Wbest 1: Load training EEGdata 2: Compute spatial filter matrix P using CSP 3: Initialize weight vectors W, population size PopSize 4:while termination condition not met max generations do 5:   for each weight vector W in population do 6:     Compute the weighted spatial filter: WP = P × Wi 7:     Extract CSP features using WP, obtain feature vector fnew 8:     Evaluate fnew using 5-fold cross-validation with an SVM classifier to obtain classification accuracy 9:     Use accuracy as the fitness of individual Wi 10:   end for 11:   Apply selection, crossover, and mutation to form a new generation 12:end while 13:Return the weight vector Wbest with the highest fitness value

The population evolution process involved the optimization of the weight gene sequence W. The initial population was generated by randomly assigning binary values to the elements of W. The weight-adjusted spatial filter matrix WP was then used to extract the spatial-domain features. The extracted features were input into an SVM classifier implemented in a MATLAB environment. Five-fold cross-validation was performed to compute the average classification accuracy, which served as the fitness value. The specific SVM parameter settings are provided in Table 1. The parameter settings of the genetic algorithm are detailed in Table 2.

**Figure 12:**
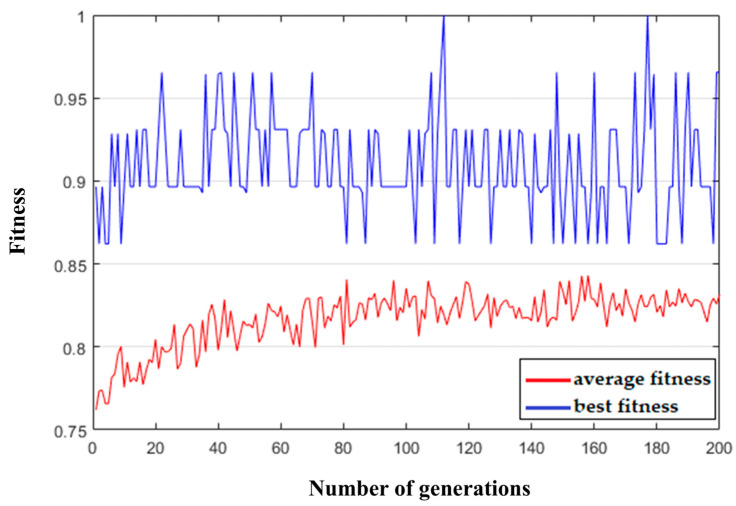
Fitness evolution curve.

The training data used in this step was the raw EEG signal A01T from subject A01 in the BCI-IV-2a dataset. After preprocessing, the left-hand and right-hand trials were selected to construct the training set for validating the proposed algorithm. During the genetic algorithm-based weight selection process, the average and best fitness values of each generation were recorded. The statistical results are shown in Figure 12. The best gene (the optimal weight sequence W) in each generation was also tracked.

**Figure 13:**
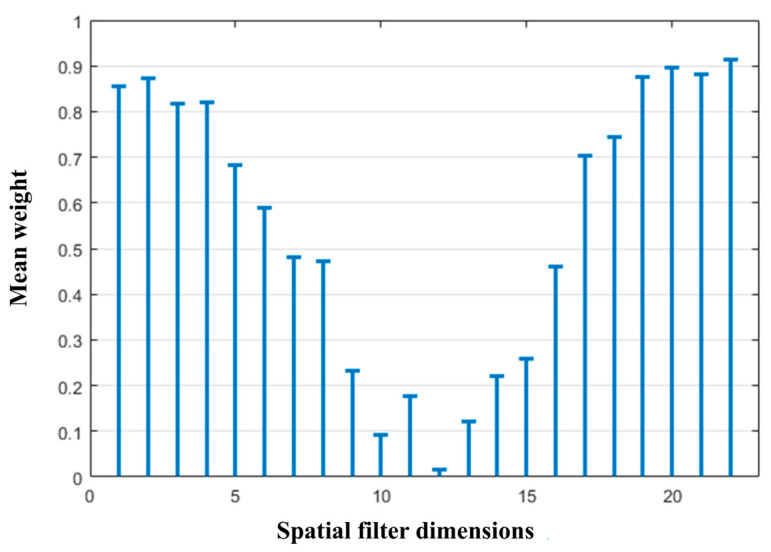
Mean optimal weight.

As shown in the figure, the average fitness value initially increased slightly as the number of generations increased and then tended to stabilize. The optimal genes from generations 100 to 200 were selected, and their binary elements were averaged, as shown in Figure 13. The weight gene sequence W corresponding to the spatial filter matrix P shows that the high-weighted features were mostly concentrated at the beginning and end of the spatial filter matrix. In contrast, the middle rows exhibited generally low weights, indicating that the corresponding features carried limited information. Therefore, to reduce the dimensionality without a significant loss of informative content, the top n and bottom n rows of the spatial filter matrix P were selected. In this step, *n* = 4 was selected, reducing the original 22-row spatial filter matrix P to 8 rows, which reduced the feature extraction complexity while retaining the most informative spatial dimensions.

#### 4.1.2. Superimposed Filter Bank Common Spatial Pattern (SFB-CSP)

The filter bank common spatial pattern (FBCSP) algorithm was first proposed in 2008 at the International Joint Conference on Neural Networks [27]. The FBCSP extends the CSP by dividing raw EEG signals into multiple frequency bands and applying the CSP separately within each band to extract the spatial-domain features, followed by feature selection and classification. This approach preserves frequency-specific information and enhances the classification accuracy.

Building on this concept, we propose an improved method, the SFB-CSP, which further enriches the spatial features with frequency-domain information. Specifically, while the FBCSP segments the 4–40 Hz range into 11 uniform 4 Hz sub-bands, the SFB-CSP targets the 0–36 Hz range using two filter bank strategies: fixed-start and sliding-window segmentation. In the fixed-start strategy, each filter shares a 0 Hz lower cutoff, with the higher cutoff increasing in 4 Hz steps, forming 9 filters of expanding bandwidths. In the sliding-window strategy, an 8 Hz bandwidth slides forward in 4 Hz steps, yielding 8 overlapping filters. The CSP is applied to each sub-band to extract the features, which are subsequently fused. The final filter in each strategy is denoted as Filter_last, and full configurations are provided in Table 3.

Excluding the overlapping bandpass filters shared by the two filter banks, a total of 16 unique bandpass filters are used. Compared to the conventional FBCSP algorithm, the proposed SFB-CSP method includes more filters and captures a broader range of frequency-domain information, making it more suitable for four-class EEG classification tasks related to lower-limb motor imagery rehabilitation. Due to the large number of filters, the number of CSP-based spatial features extracted from each filtered EEG signal results in a high-dimensional feature space. Therefore, feature extraction can focus on the most informative dimensions, typically located at the beginning and end of the spatial filter outputs.

As the filter bands obtained using the SFB-CSP method have substantial overlaps, this approach does not concatenate the spatial features across the bands. Instead, a separate SVM classifier is trained on the spatial features extracted from each frequency band. The final classification decision strategy is defined in Equation (2). The classification accuracy for each of the four spatial feature extraction methods was evaluated using both One-vs.-One (OVO) and One-vs.-Rest (OVR) strategies, with an SVM classifier applied for four-class prediction.

(2) $$L=\mathrm{argm}ax\left\{\sum_{n=1}^{N}M_{n}\left[CSP\left(F_{n}\left(E\right)\right)\right]\right\}$$

*L*—the predicted label.

*E*—the preprocessed EEG signal.

*F_n_*—a filtering operation using the nth bandpass filter.

*M_n_*—an SVM classifier for features from the nth filter.

*N*—the total number of bandpass filters.

Finally, the predicted label is determined by selecting the class with the highest number of votes among all the classifiers, denoted by the argmax operation. The pseudocode of the SFB-CSP-based classification Algorithm 2 is shown below.
**Algorithm 2.** Frequency band feature extraction and classification based on CSP and SVMData: BCI_IV_2a Result: EEG Classification 1: Load preprocessed EEGdata 2:  Initialize output list: Class 3: if Fixed Band Division is enabled then 4:   for i ∈ [0, 8] do 5:     Set start frequency = 0 Hz, end frequency = 4 × (i + 1) Hz 6:     Extract CSP features within the band 7:     Perform classification using trained SVM 8:     Store the predicted label into Class 9:   end if 10: end for 11: if Sliding Window Division is enabled then 12:   for j ∈ [1, 7] do 13:     Set start frequency = 4 × j Hz, end frequency = (4 × j) + 8 Hz 14:   Extract CSP features within the band 15:   Store the predicted label into Class 16:  end for 17: end if 18: Select the final predicted label by majority voting from Class 19: Return the final classification result

### 4.2. Multi-Domain Feature Construction and Input Optimization

EEGNet [28] is a compact CNN architecture designed to operate across various EEG-based BCI paradigms, even when only limited data is available. EEGNet builds upon traditional CNN architectures with specific optimizations and extensions, considering that standard 2D convolutional kernels struggle to effectively capture temporal features from 2D EEG signal maps. To address this, EEGNet employs 1D convolutional kernels to perform sliding convolutions over the raw temporal signals or feature maps. However, the features extracted by 1D kernels are limited in their representational capacity [29]. To address this limitation, this study proposes a CNN-based multi-class classification model using 3D EEG inputs in both the time and frequency domains. The EEG channels were rearranged into a 3D structure, and a corresponding CNN model was designed, with the inputs provided as either temporal sequences or power spectral density (PSD) sequences, to perform the multi-class classification of EEG signals.

For the time-domain processing of the raw dataset, a bandpass filter was first applied to the segmented EEG signals, with a lower cutoff frequency of 0.5 Hz and an upper cutoff of 40 Hz. Physiological artifacts were then removed using the ICA-based interactive interface described in Section 3.2. After preprocessing, the EEG signals were normalized.

The PSD provides a distribution of the power across the frequency components within a random signal. PSD analysis can be used to investigate useful information embedded in the signal. Therefore, it is also well suited for extracting frequency-domain features from EEG signals. A periodogram is a commonly used method for estimating the power spectrum. It offers computational simplicity and does not require the estimation of the autocorrelation function. For a sequence of length *N*, denoted as *x*(*n*), where 0 ≤ *n* ≤ *N* − 1, the periodogram is defined as follows:

(3) $$P_{X}\left(e^{jw}\right)=\frac{1}{N}{\left|\sum_{n=0}^{n^{2}}x\left(n\right)e^{-jw}\right|}^{2}$$

As shown in Equation (3), when *N* is large, the periodogram exhibits high variance with significant fluctuations. Conversely, when *N* is small, the frequency resolution becomes poor. To address these issues, Equation (3) was refined using the Welch method. The Welch method is a windowed averaging technique, and it is used to estimate the PSD as follows.

Let the signal *x*(*n*) of length N be represented as {*x*(0), *x*(1), …, *x*(*N* − 1)}. The signal is then divided into k segments, each consisting of *L* sampling points. When there is no overlap between the segments, the segment definition follows Equation (4). If an overlap exists between the segments, with an overlap length of *L*−*D*, then the ith segment is defined by Equations (4) and (5), where *i* = 0, 1, ..., *K* − 1 and *n* = 0, 1, ..., *L* − 1:

(4) N=k×L

(5) $$x_{i}(n)=x(n+D*i)$$

A window function *w*(*n*) is then applied to each segment to modify its periodogram. The correction process for each segment is defined by Equation (6).

(6) $${\hat{p}}_{1}\left(f\right)=\frac{1}{\sum_{n=0}^{L-1}{\left|w\left(n\right)\right|}^{2}}\sum_{n=0}^{L-1}{\left|w\left(n\right)x_{i}\left(n\right)e^{-j2\pi fn}\right|}^{2}$$

Finally, the PSD is estimated by averaging the modified periodograms across all the segments, as defined in Equation (7).

(7) $${\hat{p}}_{w}\left(f\right)=\frac{1}{k}\sum_{n=0}^{L-1}{\hat{p}}_{l}\left(f\right)$$

**Figure 14:**
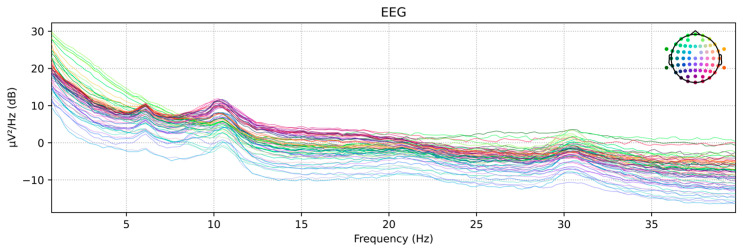
Welch PSD plot. The colored lines represent the power spectral density distributions of different EEG channels, illustrating the power variations across various frequencies for each channel.

In this study, the time-domain-preprocessed dataset was further processed in the frequency domain using the Welch PSD estimation method, and its frequency-domain features were extracted to serve as an alternative input to the proposed network model. Figure 14 shows the power spectral density maps of the raw EEG signals from subject LLS01 in the motor imagery rehabilitation dataset, extracted using the Welch PSD estimation method for each EEG channel.

**Figure 15:**
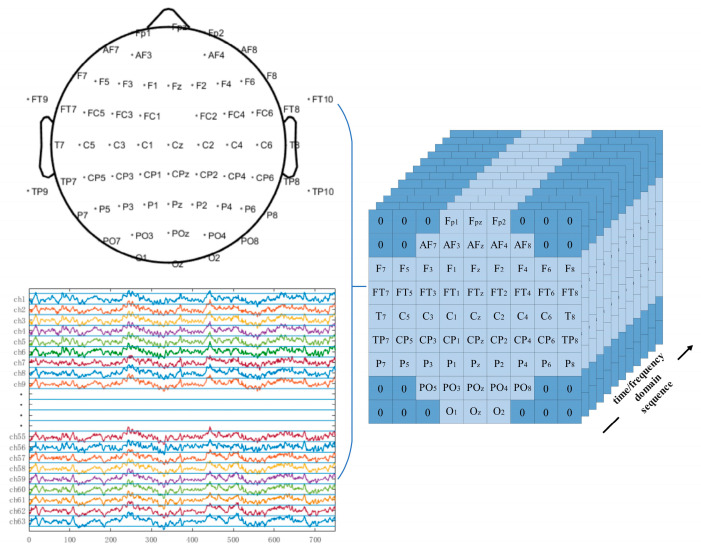
Three-dimensional EEG representations of the rehabilitation motor imagery dataset.

The previously introduced EEGNet model uses 2D EEG input representations, while the CNN model proposed in this study employed 3D EEG inputs in both the time and frequency domains. The original 2D EEG data with the shape (N,T) was spatially rearranged based on the topographic positions of the electrodes on the scalp and converted into a 3D EEG representation with the shape (W,W,T), where N is the number of EEG electrodes and T is the number of time-domain sampling points or the length of the frequency-domain feature vector. W is the side length of the reshaped spatial dimension. Positions without corresponding electrodes were zero-padded. For the motor imagery rehabilitation dataset, N = 64 and W = 9. The channel-to-grid mapping is illustrated in Figure 15.

### 4.3. Design of 3DEEG-CNN Model for Four-Class Classification

**Figure 16:**
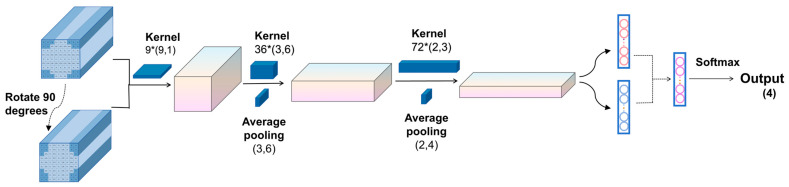
3DEEG-CNN architecture.

In conventional CNN architectures, the EEG input is typically represented as a 2D matrix of the shape (N,T), and following the EEGNet design paradigm, 1D convolutional kernels are trained to extract the temporal and spatial features in separate stages. Specifically, the first convolutional layer applies a kernel of size (1,t) to extract the temporal features from individual channels, while the second convolutional layer uses a kernel of size (n,1) to extract the spatial features across channels. Since both convolutional kernels in the 2DEEG-based design are 1D, the extraction of the temporal and spatial features is decoupled into two separate stages. For the task of four-class lower-limb motor imagery classification, the extraction of frequency-domain and spatial-domain features is particularly important. To enable the network to learn richer feature representations, this study adopted 3DEEG input formats. Two-dimensional convolutional kernels were designed to simultaneously extract both the frequency and spatial features. In addition, to further enhance frequency-domain feature extraction, the model was extended beyond time-domain 3DEEG representations to also incorporate frequency-domain 3DEEG representations, constructed from PSD features. The overall network architecture is illustrated in Figure 16.

The model took 3D EEG data as its input, with an input layer of the shape (W,W,T). The use of the time-domain 3DEEG input is considered an end-to-end classification approach. The use of the frequency-domain 3DEEG input can be regarded as a non-end-to-end classification approach. In this approach, both the training and testing sets consisted of PSD feature vectors extracted using the Welch method. These vectors were then transformed from 2D EEG representations into a 3DEEG input format according to the three-dimensional signal reconstruction scheme illustrated in Figure 15.

In conventional convolution operations, processing each row or column of 3DEEG data requires the convolution kernel to span the receptive field across all the channels, thereby making each kernel influenced by the global EEG signal during training. To address the limitations of standard convolutions, we propose a convolutional layer design that integrates 3DEEG inputs with a deep CNN architecture. The deep CNN enabled feature extraction from each individual 3DEEG channel independently, allowing each convolutional kernel to be influenced only by individual rows or columns during training, which enhanced the extraction of localized spatial features while reducing the overall computational cost.

The first convolutional layer was a depthwise convolution layer with a kernel size of (9,1) and output channel depth of W, using zero padding and a stride of 1. The activation function was ELU, and a dropout rate of 0.25 was applied. The second convolutional layer was also depthwise, with a kernel size of (3,6) and 36 output channels. It used zero padding, a stride of 1, and ELU as the activation function. A batch normalization layer was applied, along with a dropout rate of 0.25. Finally, an average pooling layer with a kernel size of (3,6) was used to reduce the feature dimensions. The third convolutional layer was again depthwise, with a kernel size of (2,3) and 72 output channels. It used zero padding, a stride of 1, and ELU as the activation function. A batch normalization layer was also included to accelerate convergence. An average pooling layer with a kernel size of (2,4) was subsequently used. A fully connected layer with 256 units was added, followed by a Softmax layer for four-class classification.

**Figure 17:**
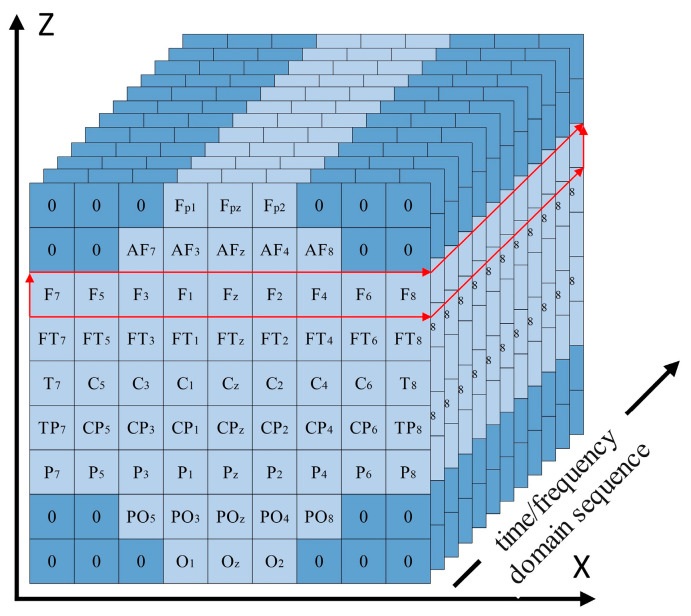
Receptive field of the first convolutional layer using a (9,1) kernel on 3DEEG data with a shape of (9,9,T).

**Figure 18:**
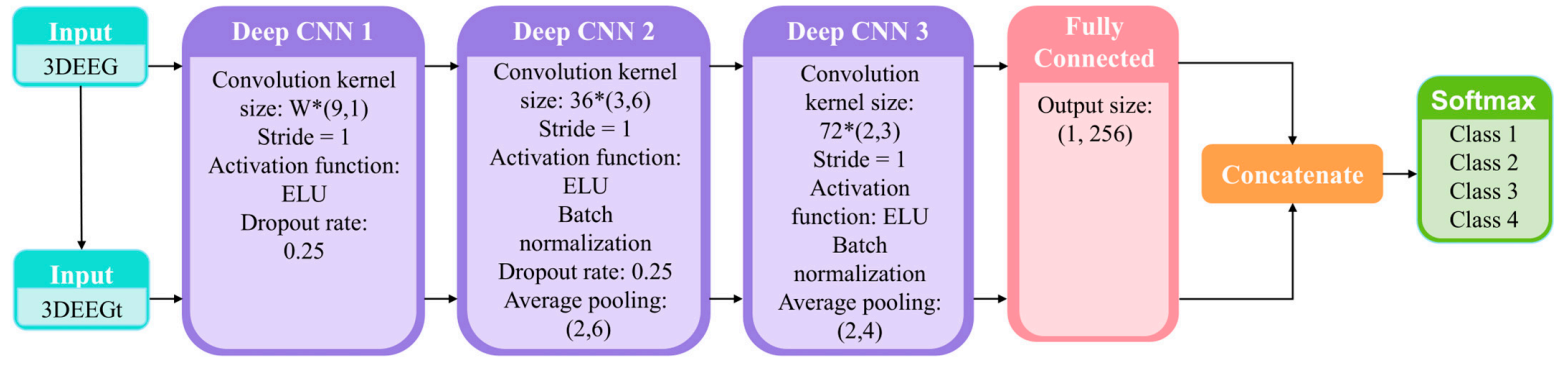
3DEEG-CNN parameter configuration.

The aforementioned network structure was capable of capturing certain features from the 3DEEG signals, but the design of the first convolutional layer still had certain limitations. For example, in the motor imagery rehabilitation dataset, the 3DEEG data had a shape of (9,9,T), and the first convolutional layer used a kernel size of (9,1) with a depth of 9, resulting in the receptive field illustrated in Figure 17, which was limited to capturing spatial features along the X-axis only. To address this limitation, the 3DEEG input matrix was rotated by 90 degrees to construct an additional input representation, thereby enabling the network to extract spatial features along the orthogonal axis without altering the overall architecture. These two input formats were processed in parallel by separate network branches, and their outputs were integrated using a concatenate layer. A Softmax layer was then used for four-class classification. This dual-input strategy enhanced the spatial feature diversity and improved the model’s decoding capability. The final network architecture is shown in Figure 18.

## 5. Experiments and Analysis of Results 

### 5.1. Results for Feature Extraction Methods

In this section, we describe how four CSP-based feature extraction methods were employed, including the conventional CSP, WC-CSP, FBCSP, and SFB-CSP. Both OVO and OVR classification strategies were used in conjunction with an SVM to evaluate their four-class classification performance. To address the sensitivity of SVM classifiers to class imbalances, we implemented strict class balancing during data preprocessing. A stratified sampling strategy ensured the equal representation of the four motor imagery categories in both the training and testing sets. Additionally, during training, 10-fold cross-validation was employed with stratified folds to preserve the class balance across all the validation sets.

Kappa values provide a more robust measure of classifier agreement compared to the accuracy, particularly in small-sample, multi-class scenarios. Table 4 and Table 5 report the Kappa values for each subject to provide a detailed view of the classifier’s performance across individual participants. For both the datasets, better classification performance was achieved for most subjects when using the features extracted using the SFB-CSP method.

**Figure 19:**
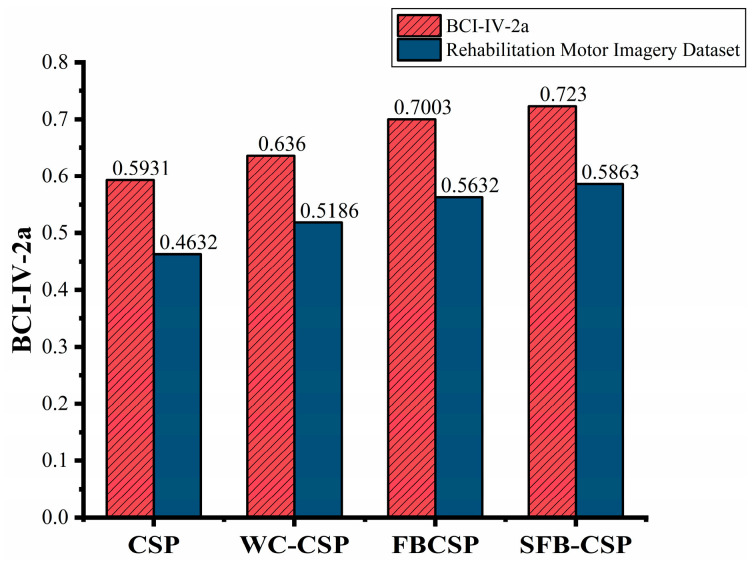
Mean accuracy comparison for four-class classification.

Figure 19 presents the average classification accuracy of the four feature extraction methods, providing an overall comparison of their performance across both datasets. The SFB-CSP method outperformed the other methods on both datasets, achieving accuracies of 72.30% on the BCI-IV-2a dataset and 58.63% on the rehabilitation motor imagery dataset. Compared to the FBCSP algorithm, the SFB-CSP improved the average four-class classification accuracy by 2.27% and 2.31% on the two datasets, respectively. This improvement can be attributed to the SFB-CSP’s ability to combine the spatial and spectral features, thereby enhancing its adaptability to multi-class tasks while offering better noise resistance and feature optimization. Additionally, the figure reveals that the classification accuracy on the rehabilitation motor imagery dataset was generally lower than that on the BCI-IV-2a dataset. This discrepancy arose from the higher similarity among motor imagery tasks in the rehabilitation dataset, whereas the tasks in the BCI-IV-2a dataset were more distinct, making class-specific features easier to discriminate.

### 5.2. Results for Classification Methods

During model training, the Adam optimizer was employed in conjunction with a cross-entropy loss function. The learning rate was set to 1e-3, and the model was trained for 200 epochs. For the rehabilitation motor imagery dataset, the input dimensions of the training and testing sets were (9,9,T), where T corresponds to the number of time-domain sampling points or the length of the PSD output in the frequency domain.

The rehabilitation motor imagery dataset comprising data from five subjects was divided into two parts: approximately 50% of the data (after the removal of corrupted segments) was used for training, and the remaining portion was used for testing. During training, 16% of the training data was further used as a validation set for monitoring the model convergence. Both the raw time-domain EEG sequences and the PSD representations of the training data were reconstructed into 3D EEG tensors. These two types of 3DEEG representations were then used as dual inputs to train the model. Subsequently, the corresponding 3DEEG representations from the testing set were used to evaluate the model performance.

Among the models evaluated on the dataset comprising data from five subjects, the PSD-3DEEG-CNN achieved the best performance, with an average accuracy of 62.68%, a maximum accuracy of 66.32%, and an average Kappa score of 0.531. This was followed by the time-domain 3DEEG-CNN, which yielded an average accuracy of 58.01%, a peak accuracy of 60.71%, and an average Kappa score of 0.475. In contrast, the conventional EEGNet model attained a lower average accuracy of 55.06% and an average Kappa score of only 0.444, indicating inferior performance compared to the two proposed 3DEEG-based models. Moreover, a comparison between the two proposed 3DEEG-CNN variants revealed that the frequency-domain 3DEEG model based on Welch’s PSD analysis performed better when decoding signals in lower-limb rehabilitation motor imagery tasks, which are known to exhibit low inter-class separability. Detailed results are presented in Table 6.

**Figure 20:**
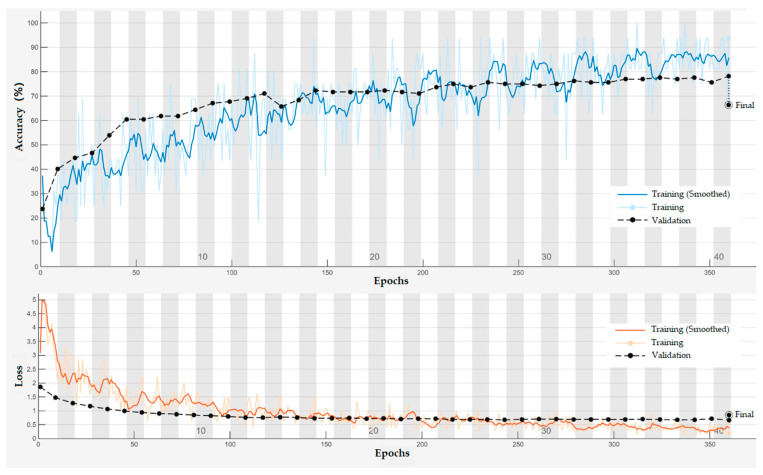
Training and validation curves of the PSD-3DEEG-CNN model over 360 epochs on the rehabilitation motor imagery dataset. The validation accuracy and loss were computed using a held-out validation split during training. The final accuracy of 66.32% was obtained on the complete test set.

Figure 20 presents the training and validation accuracy and loss curves of the PSD-3DEEG-CNN model over 360 epochs. The training loss decreased rapidly during the first 80 epochs and stabilized thereafter, while the training accuracy steadily improved despite moderate fluctuations. The validation accuracy, evaluated every nine epochs, exhibited a relatively flat trend after epoch 150, suggesting convergence. The final test accuracy of 66.32% suggests that the proposed network is capable of learning discriminative EEG representations for multi-class motor imagery classification, despite the inherent challenges of low signal-to-noise ratios and inter-class similarity in lower-limb EEG data.

**Figure 21:**
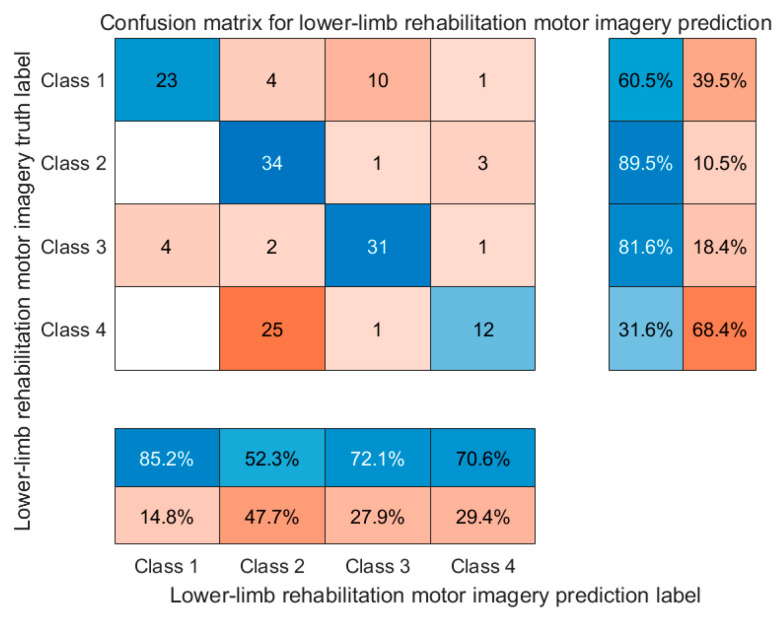
Normalized confusion matrix of the PSD-3DEEG-CNN model for four-class lower-limb motor imagery classification. Each row represents the true class, and each column represents the predicted class. Class labels: Class 1—left leg flexion; Class 2—left leg extension; Class 3—right leg flexion; Class 4—right leg extension.

To further examine the model’s class-wise performance, a confusion matrix of the final predictions on the test set is presented in Figure 21.

This study achieved promising classification results on a self-collected dataset, providing preliminary evidence for the effectiveness of the proposed feature extraction methods and deep learning model in multi-class motor imagery tasks. We attribute this excellent result to three factors: (1) In the feature extraction stage, the SFB-CSP method integrated spatial and spectral information by applying a denser subdivision of sub-bands. This enhanced the sensitivity to subtle EEG variations and improved both the separability and robustness of the extracted features. (2) In the classification stage, the proposed 3DEEG-CNN leveraged the topological arrangement of the EEG channels for three-dimensional modeling, significantly improving the model’s ability to capture local spatial patterns. When the frequency-domain features extracted using Welch’s PSD estimation method were also used, the model received a more stable and discriminative spectral representation as its input. (3) The incorporation of an automated artifact removal tool reduced the contamination from EMG and ocular artifacts, thereby increasing the signal-to-noise ratio of the training data and providing a cleaner foundation for model learning.

### 5.3. Computational Complexity and Real-Time Feasibility

While the proposed 3DEEG-CNN and SFB-CSP methods significantly improved the classification performance on both the rehabilitation and public datasets, their computational complexity raises concerns regarding their feasibility in real-time or embedded BCI systems.

The 3DEEG-CNN model employs a multi-branch deep architecture that includes several convolutional and pooling layers. This design enables the extraction of spatial features across dual axes but inevitably increases the model’s parameter size, inference latency, and memory consumption. The dual-input strategy involving coordinate transformation further increases the forward-pass computation. Although these design choices are justified as they improve the decoding accuracy, they may pose a challenge for deployment in latency-sensitive or low-resource environments, such as wearable neuro-rehabilitation devices.

Although the precise inference time was not evaluated in this study, preliminary profiling suggested a non-negligible computational cost. This limitation is especially relevant for real-time or embedded BCI scenarios, where the latency and efficiency are critical. Therefore, future work will include systematic profiling and real-time simulation testing to better assess the deployability of the proposed methods.

In parallel, the SFB-CSP method, while lightweight in inference, introduces a substantial preprocessing overhead. It requires filter bank decomposition followed by CSP feature extraction for each sub-band, which involves matrix operations and eigenvalue decomposition for every trial. This multi-step pipeline may be computationally demanding when scaled to continuous or online classification scenarios.

While these latencies are acceptable in offline research settings, real-time applications would require further optimization. Future work will focus on three directions to enhance the practicality of the proposed methods: (1) model pruning and quantization to reduce the CNN model’s size and latency, (2) adaptive sub-band selection to reduce redundancy in the SFB-CSP, and (3) exploring lightweight hybrid models that balance performance and efficiency. These efforts aim to bridge the gap between academic performance and real-world deployability in closed-loop BCI systems for motor rehabilitation.

## 6. Conclusions

This study systematically investigated the multi-class classification of lower-limb MI-EEG signals for rehabilitation. An automated artifact removal tool was developed using the MNE toolbox and PyQt5, enabling the efficient and accurate elimination of common EEG artifacts. To enhance feature representation, this study proposed novel spatial–spectral feature extraction methods, including the weighted CSP and SFB-CSP, which integrated spatial and frequency-domain information. Given the high inter-class similarity and low separability of EEG patterns associated with different lower-limb motor imagery tasks in the self-collected dataset, we further designed a convolutional neural network model that took 3D EEG representations as its input. By separately modeling the temporal and frequency characteristics, the network’s ability to capture both the spatial topography and spectral dynamics of EEG signals was significantly improved. The experimental results on the self-collected lower-limb MI dataset demonstrate that the proposed approach substantially outperformed the conventional EEGNet baseline, validating its effectiveness and superiority in complex multi-class BCI scenarios.

The four-class classification approach proposed in this study for lower-limb motor imagery rehabilitation tasks achieved higher accuracy compared to several existing methods. Nevertheless, certain limitations remain, and further improvements are necessary in future research.

1. Future work will focus on developing an end-to-end deep neural network that unifies the traditionally decoupled processes of feature extraction and classification into a single architecture, enabling direct mapping from raw EEG signals to class outputs.
2. Given that the classification performance is highly dependent on the quality of the input features, future studies will explore the incorporation of more diverse feature types or the design of more effective feature fusion strategies to enhance complementarity and information representation.
3. The upper bound of the classification accuracy may still be constrained by the capacity of the current models. Therefore, future efforts will aim to design more advanced AI architectures capable of further improving the classification performance under the same feature conditions.

## Figures and Tables

**Table 1:** Parameters of the SVM classifier.

Parameter	Value
Kernel Function	RBF
Kernel Scale	0.783
Regularization	0.922

**Table 2:** Parameters of genetic algorithm.

Parameter	Value
Population Size	50
Number of Generations	200
Crossover Rate	0.5
Mutation Rate	0.06

**Table 3:** Different bandpass filters.

Filter Bank Type	Filter 1	Filter 2	Filter 3	…	Last Filter
Fixed-Start Band Segmentation	0–4 Hz	0–8 Hz	0–12 Hz	…	0–36 Hz
Equally Spaced Sliding Segmentation	0–8 Hz	4–12 Hz	8–16 Hz	…	28–36 Hz

**Table 4:** Kappa values for different CSP-based feature extraction methods on the rehabilitation MI dataset.

Subject	CSP		WC-CSP		FBCSP		SFB-CSP	
	OVO	OVR	OVO	OVR	OVO	OVR	OVO	OVR
A01	0.433	0.365	0.495	0.469	0.592	0.627	0.622	**0.639**
A02	0.377	0.389	0.342	0.399	0.552	0.539	0.603	**0.609**
A03	0.484	0.455	0.554	0.532	0.672	0.627	0.662	**0.677**
A04	0.376	0.326	0.386	0.33	0.501	0.486	**0.65**	0.641
A05	0.59	0.531	0.637	0.605	**0.693**	0.672	0.631	0.647
A06	0.349	0.402	0.425	0.433	0.61	**0.622**	0.62	0.601
A07	0.495	0.421	0.503	0.485	0.599	0.603	0.671	**0.693**
A08	0.585	0.476	0.627	0.573	0.654	0.62	0.683	**0.705**
A09	0.312	0.291	0.328	0.246	0.532	0.55	0.649	**0.662**

**Table 5:** Kappa values for different CSP-based feature extraction methods on the BCI-IV-2a dataset.

Subject	CSP		WC-CSP		FBCSP		SFB-CSP	
	OVO	OVR	OVO	OVR	OVO	OVR	OVO	OVR
LLS01	0.284	0.277	0.299	0.243	0.32	0.337	0.374	**0.389**
LLS02	0.297	0.305	0.351	0.328	**0.49**	0.423	0.472	0.485
LLS03	0.349	0.366	0.392	0.386	0.423	0.475	**0.465**	0.452
LLS04	0.331	0.324	0.345	0.336	0.375	0.356	0.397	**0.421**
LLS05	0.312	0.33	0.356	0.342	0.403	0.485	0.544	**0.583**

**Table 6:** Classification results for the rehabilitation MI dataset.

Subject	Time-Domain 3DEEG-CNN		PSD-3DEEG-CNN		EEGNET	
	Accuracy (%)	Kappa	Accuracy (%)	Kappa	Accuracy (%)	Kappa
LLS01	52.66	0.408	**59.29**	**0.49**	48.98	0.382
LLS02	60.24	0.504	**65.85**	**0.573**	54.3	0.43
LLS03	58.57	0.482	**60.28**	**0.502**	57.14	0.465
LLS04	57.86	0.473	**61.67**	**0.52**	60.34	0.509
LLS05	60.71	0.508	**66.32**	**0.582**	54.52	0.432
Average	58.01	0.475	**62.68**	**0.531**	55.06	0.444

## Data Availability

The data presented in this study are available from the corresponding author, X.P., on reasonable request.
